# 脐带血辅助单倍体外周血干细胞移植与无关供者外周血干细胞移植治疗恶性血液病的疗效比较

**DOI:** 10.3760/cma.j.cn121090-20230928-00149

**Published:** 2024-02

**Authors:** 霞 马, 焱 陈, 弋 刘, 婷婷 程, 旭 陈, 枞 曾, 娟 华, 诗宇 王, 雅靖 徐

**Affiliations:** 中南大学湘雅医院血液内科，国家老年疾病临床医学研究中心（湘雅医院），湖南省血液肿瘤研究中心，长沙 410008 Department of Hematology, Xiangya Hospital, Central South University; National Clinical Research Center for Geriatric Diseases (Xiangya Hospital); Hunan Clinical Medical Research Center of Hematologic Neoplasms, Changsha 410008, China

**Keywords:** 单倍体造血干细胞移植, 脐带血, 无关供者造血干细胞移植, Haploidentical hematopoietic stem cell transplantation, Umbilical cord blood, Unrelated donor hematopoietic stem cell transplantation

## Abstract

**目的:**

比较脐带血辅助单倍体外周血干细胞移植（haplo-cord-PBSCT）与无关供者外周血干细胞移植（UD-PBSCT）治疗恶性血液病的疗效。

**方法:**

对中南大学湘雅医院2016年1月至2021年12月的104例接受haplo-cord-PBSCT和52例接受UD-PBSCT）的恶性血液病患者进行回顾性分析。

**结果:**

①haplo-cord-PBSCT和UD-PBSCT组中性粒细胞中位植入时间分别为13（9～22）d、13（10～24）d（*P*＝0.834），血小板植入中位时间分别为15（7～103）d、14（8～38）d（*P*＝0.816）。haplo-cord-PBSCT组和UD-PBSCT组移植后30 d中性粒细胞累积植入率均为100.0％（*P*＝0.314），移植后100 d血小板累积植入率分别为95.2％（95％*CI* 88.3％～98.1％）、100.0％（*P*＝0.927）。移植后30 d两组患者均达到完全供者嵌合状态，未发生脐带血干细胞植入。②haplo-cord-PBSCT组与UD-PBSCT组移植后100 d内Ⅱ～Ⅳ度急性GVHD的累积发生率分别为29.1％（95％*CI* 20.1％～38.1％）、28.8％（95％*CI* 17.2％～41.6％（*P*＝0.965），Ⅲ/Ⅳ度急性GVHD的累积发生率分别为7.8％（95％*CI* 3.6％～14.0％）、9.6％（95％*CI* 3.5％～19.5％）（*P*＝0.725）。haplo-cord-PBSCT组与UD-PBSCT组2年慢性GVHD的累积发生率分别为45.3％（95％*CI* 36.1％～56.1％）、35.1％（95％*CI* 21.6％～44.1％）（*P*＝0.237），移植后2年重度慢性GVHD的累积发生率分别为13.6％（95％*CI* 7.6％～21.3％）、12.9％（95％*CI* 5.1％～24.3％）（*P*＝0.840）。③haplo-cord-PBSCT组、UD-PBSCT组移植后2年CIR分别为12.8％（95％*CI* 7.0％～20.5％）、10.0％（95％*CI* 3.6％～20.2％）（*P*＝0.341），NRM分别为14.7％（95％*CI* 8.4％～22.6％）、16.2％（95％*CI* 7.4％～28.0％）（*P*＝0.681）。④haplo-cord-PBSCT组、UD-PBSCT组移植后2年OS率分别为82.2％（95％*CI* 74.8％～90.3％）、75.5％（95％*CI* 64.2％～88.7％）（*P*＝0.276），2年DFS率分别为69.9％（95％*CI* 61.2％～79.8％）、73.8％（95％*CI* 62.4％～87.3％）（*P*＝0.551），2年无GVHD无复发生存（GRFS）率分别为55.3％（95％*CI* 44.8％～64.8％）、64.7％（95％*CI* 52.8％～79.3％）（*P*＝0.284）。

**结论:**

haplo-cord-PBSCT与UD-PBSCT治疗恶性血液病具有相似的疗效和安全性，可作为恶性血液病的替代治疗方案。

## Abstract (en)

**Objective:**

To assess the efficacy of cord blood-assisted haploid peripheral blood stem cell transplantation （haplo-cord-PBSCT） versus unrelated donor peripheral blood stem cell transplantation （UD-PBSCT） in the treatment of malignant hematological diseases.

**Methods:**

A retrospective analysis was performed on one hundred and four patients with malignant hematological diseases who underwent haplo-cord-PBSCT and fifty-two patients who underwent UD-PBSCT at Xiangya Hospital of Central South University between January 2016 and December 2021.

**Results:**

①The median implantation time for neutrophils in the haplo-cord-PBSCT and UD-PBSCT groups was 13（9–22） days and 13（10–24） days, respectively （*P*＝0.834）, whereas the median implantation time for platelets was 15（7–103） days and 14 （8–38） days, respectively （*P*＝0.816）. The cumulative implantation rate of neutrophils at 30 days after transplantation in the haplo-cord-PBSCT group and the UD-PBSCT group was 100％（*P*＝0.314）, and the cumulative platelet implantation rate at 100 days after transplantation was 95.2％ （95％*CI* 88.3％–98.1％） and 100％（*P*＝0.927）, respectively. 30 days after transplantation, both groups of patients achieved complete donor chimerism, and no umbilical cord blood stem cells were implanted. ②The cumulative incidence rates of grade Ⅱ-Ⅳ acute GVHD within 100 days after transplantation in the haplo-cord-PBSCT group and the UD-PBSCT group were 29.1％ （95％*CI* 20.1％–38.1％） and 28.8％ （95％*CI* 17.2％–41.6％（*P*＝0.965）, respectively. The cumulative incidence rates of grade Ⅲ/Ⅳ acute GVHD were 7.8％ （95％ *CI* 3.6％–14.0％） and 9.6％ （95％*CI* 3.5％–19.5％）（*P*＝0.725）. The cumulative incidence rates of 2-year chronic GVHD in the haplo-cord-PBSCT group and the UD-PBSCT group were 45.3％ （95％*CI* 36.1％–56.1％） and 35.1％ （95％*CI* 21.6％–44.1％）, respectively （*P*＝0.237）. The cumulative incidence rates of severe chronic GVHD at 2 years after transplantation were 13.6％ （95％*CI* 7.6％–21.3％） and 12.9％ （95％*CI* 5.1％–24.3％）, respectively （*P*＝0.840）. ③The 2-year CIR after transplantation in the haplo-cord-PBSCT group and UD-PBSCT group were 12.8％ （95％CI 7.0％–20.5％） and 10.0％ （95％*CI* 3.6％–20.2％）, respectively （*P*＝0.341）, and the NRM were 14.7％ （95％ *CI* 8.4％–22.6％） and 16.2％ （95％*CI* 7.4％–28.0％）, respectively （*P*＝0.681）. ④The 2-year OS rates in the haplo-cord-PBSCT and UD-PBSCT groups after transplantation were 82.2％ （95％*CI* 74.8％–90.3％） and 75.5％ （95％*CI* 64.2％-88.7％）, respectively （*P*＝0.276）. The 2-year DFS rates were 69.9％ （95％ *CI* 61.2％–79.8％） and 73.8％ （95％*CI* 62.4％–87.3％）, respectively （*P*＝0.551）. The 2-year rates of GVHD-free/recurrence-free survival （GRFS） were 55.3％ （95％*CI* 44.8％–64.8％） and 64.7％ （95％*CI* 52.8％–79.3％）, respectively （*P*＝0.284）.

**Conclusion:**

The findings of this study indicate that haplo-cord-PBSCT and UD-PBSCT have comparable efficacy and safety in the treatment of malignant hematological diseases and can be used as an alternative treatment options.

无关供者造血干细胞移植（UD-HSCT）等待时间相对较长，等待期间可能出现疾病进展，并且移植后难以获得供者淋巴细胞或干细胞。既往研究表明，单倍体造血干细胞移植（haplo-HSCT）可取得与UD-HSCT相似的生存疗效[1]–[2]，但移植后移植物抗宿主病（GVHD）、感染等并发症发生率以及非复发死亡率（NRM）较高 [1],[3]–[4]。与单纯haplo-HSCT或脐带血移植相比，haplo-HSCT联合脐带血移植能够促进造血恢复、降低GVHD发生率和复发率[5]–[6]。我们先前的研究结果表明，脐带血辅助单倍体外周血干细胞移植（haplo-cord-PBSCT）可获得与同胞全相合外周血干细胞移植（MSD- PBSCT）相似的疗效，GVHD发生率和NRM也无显著差异[7]。本研究中，我们对本中心haplo-cord-PBSCT与无关供者外周血干细胞移植（UD-PBSCT）治疗恶性血液病的疗效进行回顾性分析。

## 病例与方法

一、研究设计

本研究共纳入2016年1月至2021年12月于中南大学湘雅医院行异基因造血干细胞移植（allo-HSCT）的恶性血液病患者156例，其中haplo-cord-PBSCT组104例，UD-PBSCT组52例。入选标准：①参照NCCN和WHO标准纳入年龄小于60岁的恶性血液病患者，包括急性髓系白血病（AML）（除急性早幼粒细胞白血病）、急性淋巴细胞白血病（ALL）和骨髓增生异常综合征（MDS）患者；②有allo-HSCT适应证；③非血缘供者均为高分辨人类白细胞抗原（HLA）10/10相合，即HLA-A、-B、-C、-DRB1和-DQB1 5个位点10个等位基因完全相同；④接受清髓性预处理；⑤首次allo-HSCT。本研究经中南大学湘雅医院研究与伦理委员会批准，患者或其法定监护人均知情同意。

二、HLA配型和供者选择

所有供患者及脐带血均接受HLA高分辨配型位点检测，包括HLA-A、-B、-C、-DRB1、-DQB1。脐带血的入选标准如下：①与患者配型的脐带血结果HLA ≥ 5/10，且每个配型位点至少有1个相合；②解冻后单个核细胞（MNC）数量不低于1×10^7^/kg（患者体重），若脐带血配型相合位点数相同，优先选择血型相合者。所有患者移植前均行HLA抗体检测，供者特异性抗体（DSA）均为阴性或弱阳性（平均荧光强度<4 000），弱阳性患者均未进行特殊处理。脐带血DSA均为阴性。

三、预处理方案

本研究所有患者均使用改良BuCy方案进行预处理：阿糖胞苷4 g·m^−2^·d^−1^，−8 d、−7 d；白消安3.2 mg·kg^−1^·d^−1^，−6 d～−4 d；环磷酰胺1.8 g·m^−2^·d^−1^，−3 d、−2 d；司莫司汀250 mg/m^2^，−2 d；兔抗人胸腺细胞球蛋白（rATG）2.5 mg·kg^−1^·d^−1^，−3 d～−1 d。

四、供者干细胞的采集与输注

所有供者均使用G-CSF（7.5～10 µg·kg^−1^·d^−1^）动员外周血造血干细胞。haplo-cord-PBSCT组在第1天输注外周血干细胞，第2天输注脐带血，脐带血的输注时间与外周血干细胞输注结束时间至少间隔12 h，并且输注的脐带血MNC细胞数均为1×10^7^/kg（患者体重）。UD-PBSCT组在第1天输注外周造血干细胞，必要时第2天再次输注。

五、GVHD预防

所有患者均采用环孢素A（CsA）、霉酚酸酯（MMF）、短程甲氨蝶呤（MTX）预防GVHD。−9 d开始以CsA 2.5 mg·kg^−1^·d^−1^静滴，维持血浓度200～250 µg/L。MMF于−9 d开始口服。若无GVHD发生，UD-PBSCT组中性粒细胞植入时MMF剂量减半，+30 d停用；haplo-cord-PBSCT组+30 d MMF剂量减半，+45 d～+60 d停用。所有患者均于+1 d、 +3 d、+6 d分别予以MTX 15、10、10 mg/m^2^，haplo-cord-PBSCT组在+11 d加用1次MTX 10 mg/m^2^。

发生Ⅱ度及以上急性GVHD时首选甲泼尼龙静脉滴注治疗，效果不佳者选择巴利昔单抗、芦可替尼或其他二线治疗方案。

六、预防感染和移植后监测

患者在预处理前3～5 d使用制霉菌素和庆大霉素进行移植前肠道准备。在预处理和免疫抑制期间给予预防性抗真菌治疗和抗病毒治疗，并予以复方磺胺甲唑预防耶氏肺孢子菌感染。所有患者移植后规律行骨髓穿刺检查评估骨髓缓解状态和嵌合情况，必要时行腰穿检查及鞘内注射化疗药物。

七、终点事件及定义

本研究主要关注总生存（OS）、无病生存（DFS）、累积复发率（CIR）、NRM、急性GVHD、慢性GVHD及无GVHD无复发生存（GRFS）。急性GVHD和慢性GVHD的诊断和分级参照文献[8]–[9]，AML/MDS和ALL诊断标准参照WHO标准，危险分层参照ELN/NCCN标准和国际预后评分系统。

八、随访

本研究采用查阅住院/门诊病例和电话随访模式，随访截至2022年9月30日。

九、统计学处理

采用SPSS26.0和R软件计算，两组患者基线特征比较采用Mann-Whitney *U*检验对连续变量进行比较，采用*χ*^2^检验对分类资料进行比较。采用Kaplan-Meier法计算OS、DFS、GRFS，并采用Log-rank检验进行比较。采用竞争风险模型（累积发病率法）计算中性粒细胞植入率、血小板植入率、GVHD发生率、CIR和NRM，以Gray's检验进行组间比较。采用Cox回归模型进行单因素分析和OS、DFS和GRFS的多因素分析，采用有竞争风险的回归模型对造血干细胞植入率、CIR、NRM和GVHD进行多因素分析，检验均为双侧，*P*值<0.05表示差异有统计学意义。

## 结果

一、患者的基本特征

患者的基本特征见表1。haplo-cord-PBSCT组高危患者占比高于UD-PBSCT组（62.5％对30.8％，*P*<0.001），随访期间存活患者中haplo-cord-PBSCT组、UD-PBSCT组的中位随访时间分别为28.70（2.10～58.90）、36.30（4.13～81.70）个月。haplo-cord-PBSCT组单个核细胞输注量高于UD-PBSCT组（10.25×10^8^/kg对8.69×10^8^/kg，*P*<0.001）。

**表1:** 脐血辅助单倍体外周血干细胞移植（Haplo-cord-PBSCT）与无关供者外周血干细胞移植（UD-PBSCT）组恶性血液病患者及供者的临床资料

指标	Haplo-cord-PBSCT组（104例）	UD-PBSCT组（52例）	*P*值
性别［例（%）］			1.000
男	56（53.8）	28（53.8）	
女	48（46.2）	24（46.2）	
年龄［岁，*M*（范围）］	28（7~56）	30（14~57）	0.494
诊断［例（%）］			0.158
AML/MDS	62（59.6）	37（71.8）	
ALL	42（40.4）	15（28.2）	
危险分层［例（%）］			
所有患者			<0.001
标危/中危	39（37.5）	36（48）	
高危	65（62.5）	16（30.8）	
AML/MDS			0.002
中危	22（35.5）	25（67.6）	
高危	40（64.5）	12（32.4）	
ALL			0.029
标危	17（40.5）	11（73.3）	
高危	25（59.5）	4（26.7）	
移植前疾病状态［例（%）］			
所有患者			0.416
CR	91（87.5）	43（82.7）	
其他	13（12.5）	9（17.3）	
AML/MDS			0.216
CR1	34（54.8）	22（59.5）	
≥CR2	20（32.3）	7（18.9）	
其他	8（12.9）	8（21.6）	
ALL			0.009
CR1	16（38.1）	12（80.0）	
≥CR2	21（50.0）	1（6.7）	
其他	5（11.9）	2（13.3）	
移植前MRD状态［例（%）］			0.605
阳性	8（8.8）	5（11.6）	
阴性	83（91.2）	38（88.4）	
供患者血型［例（%）］			0.025
相合	70（67.3）	22（42.3）	
大不合	10（9.6）	11（21.2）	
小不合	20（19.2）	16（30.8）	
双向不合	4（3.9）	3（5.8）	
供患者性别［例（%）］			0.041
男供男	34（32.7）	24（46.2）	
男供女	37（35.6）	22（42.3）	
女供男	21（20.2）	5（9.6）	
女供女	12（11.5）	1（1.9）	
外周血MNC［×10^8^/kg，*M*（范围）］	10.25（4.63~19.30）	8.69（1.74~19.40）	<0.001
外周血CD34^+^细胞［×10^6^/kg，*M*（范围）］	5.93（1.61~14.12）	5.35（0.96~12.32）	0.060
脐带血MNC（×10^7^/kg）	1.0	/	
脐带血CD34^+^细胞［×10^5^/kg，*M*（范围）］	0.24（0.08~0.77）	/	
确诊至移植时间［月，*M*（范围）］	6.35（2.13~44.33）	7.43（3.56~31.73）	0.003

注 AML：急性髓系白血病；ALL：急性淋巴细胞白血病；MDS：骨髓增生异常综合征；CR1：第一次完全缓解；CR2：第二次完全缓解；MRD：微小残留病；MNC：单个核细胞；/：不适用

二、造血恢复和植入情况

haplo-cord-PBSCT和UD-PBSCT组中性粒细胞中位植入时间分别为13（9～22）d、13（10～24）d（*P*＝0.834），血小板植入中位时间分别为15（7～103）d、14（8～38）d（*P*＝0.816）。haplo-cord-PBSCT组和UD-PBSCT组移植后30 d中性粒细胞累积植入率均为100.0％（*P*＝0.314），移植后100 d血小板累积植入率分别为95.2％（95％*CI* 88.3％～98.1％）、100.0％（*P*＝0.927）。移植后30 d两组患者均达到完全供者嵌合状态，未发生脐血干细胞植入。

三、GVHD

haplo-cord-PBSCT组与UD-PBSCT组移植后100 d内Ⅱ～Ⅳ度急性GVHD的累积发生率分别为29.1％（95％*CI* 20.1％～38.1％）、28.8％（95％*CI* 17.2％～41.6％（*P*＝0.965），Ⅲ/Ⅳ度急性GVHD的累积发生率分别为7.8％（95％*CI* 3.6％～14.0％）、9.6％（95％*CI* 3.5％～19.5％）（*P*＝0.725）。haplo-cord-PBSCT组与UD-PBSCT组2年慢性GVHD的累积发生率分别为45.3％（95％*CI* 36.1％～56.1％）、35.1％（95％*CI* 21.6％～44.1％）（*P*＝0.237），移植后2年重度慢性GVHD的累积发生率分别为13.6％（95％*CI* 7.6％～21.3％）、12.9％（95％*CI* 5.1％～24.3％）（*P*＝0.840）。

四、复发及NRM

haplo-cord-PBSCT组、UD-PBSCT组移植后2年CIR分别为12.8％（95％*CI* 7.0％～20.5％）、10.0％（95％*CI* 3.6％～20.2％）（*P*＝0.341），2年NRM分别为14.7％（95％*CI* 8.4％～22.6％）、16.2％（95％*CI* 7.4％～28.0％）（*P*＝0.681）。

五、生存情况

至随访终点，haplo-cord-PBSCT组17例患者死亡，UD-PBSCT组12例患者死亡，死亡原因详见表2。haplo-cord-PBSCT组、UD-PBSCT组移植后2年OS率分别为82.2％（95％*CI* 74.8％～90.3％）、75.5％（95％*CI* 64.2％～88.7％）（*P*＝0.276）（图1A）。2年DFS率分别为69.9％（95％*CI* 61.2％～79.8％）、73.8％（95％*CI* 62.4％～87.3％）（*P*＝0.551）（图1B），2年GRFS率分别为55.3％（95％*CI* 44.8％～64.8％）、64.7％（95％*CI* 52.8％～79.3％）（*P*＝0.284）（图1C）。

**表2:** 接受脐带血辅助单倍体外周血干细胞移植（haplo-cord-PBSCT）与无关供者外周血干细胞移植（UD-PBSCT）恶性血液病患者的主要死亡原因分布（例）

组别	例数	死亡原因				
		复发	感染	GVHD	其他	合计
Haplo-cord-PBSCT组	104	1	7	7	2	17
UD-PBSCT组	52	4	4	2	2	12

**图1:**
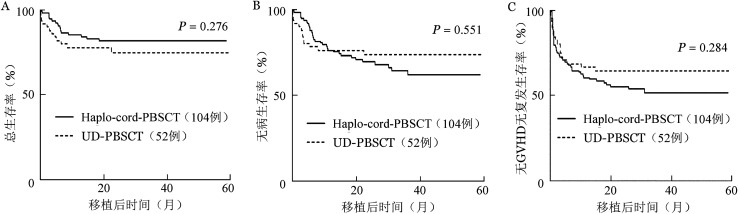
接受脐带血辅助单倍体外周血干细胞移植（Haplo-cord-PBSCT）与无关供者外周血干细胞移植（UD-PBSCT）恶性血液病患者的移植后生存曲线 A 总生存；B 无病生存；C 无移植物抗宿主病（GVHD）无复发生存

## 讨论

对于缺乏HLA完全相合的同胞或无关供者的恶性血液病患者，haplo-HSCT已经成为重要的替代治疗方法。既往研究表明，haplo-HSCT取得了与无关或同胞全相合造血干细胞移植（MSD-HSCT）相似的OS和DFS，但植入延迟、相对较高的GVHD发生率、NRM和感染等仍是影响haplo-HSCT患者预后的重要因素[1],[10]–[12]。在本研究中，haplo-cord-PBSCT组与UD-PBSCT组相比，生存情况无显著性差异，同时在造血干细胞植入、GVHD、NRM和CIR等方面的差异也无统计学意义。

既往研究表明haplo-HSCT的造血重建慢于较UD-HSCT，Luo等[13]回顾性研究结果显示，基于G-CSF/ATG方案的haplo-HSCT组的移植后15 d中性粒细胞和30 d血小板植入率均低于UD-HSCT组（78.6％对97.4％，*P*<0.05；97.5％对100％，*P*<0.05）。Yu等[14]研究结果显示，在接受造血干细胞移植治疗急性白血病患者中，G-CSF/ATG方案单倍体外周血干细胞联合骨髓移植组的中性粒细胞及血小板植入的中位时间均晚于UD-HSCT组。Gooptu等[15]研究显示，PTCY方案单倍体外周血干细胞或骨髓移植患者的移植后30 d中性粒细胞植入率与UD-HSCT组相比无明显差异，但移植后100 d血小板植入率较低（88％对95％）。本研究中haplo-cord-PBSCT组与UD-PBSCT组中性粒细胞和血小板植入的中位时间及累积植入率差异均无统计学意义，可能与脐带血有助于促进造血重建及预防原发性植入功能不良有关。脐带血造血干细胞的再生能力较骨髓或外周血干细胞更强，原始造血祖细胞和多能集落形成细胞等造血祖细胞的比例较高，脐带血CD34^+^祖细胞的增殖能力和多向分化潜能也明显高于骨髓和外周血干细胞[16]–[17]。因此，脐带血的加入可能促进单倍体外周血干细胞移植后造血系统的重建，具体的作用机制，有待于进一步研究。本研究中的haplo-cord-PBSCT组干细胞来源为纯外周血干细胞，相较于骨髓移植，外周干细胞移植的植入时间更短，植入失败率较低[18]–[19]。

本研究中haplo-cord-PBSCT组患者均获得单倍体供者完全嵌合，未发生脐血造血干细胞植入，与其他脐带血辅助haplo-HSCT研究不同[20]–[21]，考虑可能原因为我们输注的脐带血单个核细胞数较低（均为1×10^7^/kg），且脐带血输注时间与外周血干细胞输注结束时间至少间隔12 h。

GVHD是影响haplo-HSCT患者预后的主要因素之一。Huang等[22]研究发现基于G-CSF/ATG方案的单倍体外周血联合骨髓移植患者Ⅱ～Ⅳ度急性GVHD发生率高于UD-HSCT组（47％对31％，*P*＝0.033），两组慢性GVHD发生率无明显差异。Yu等[14]的研究也获得类似的结果。Lu等[1]的研究表明，基于G-CSF/ATG方案单倍体外周血联合骨髓移植治疗恶性血液病患者的Ⅱ～Ⅳ度、Ⅲ/Ⅳ度急性GVHD和慢性GVHD发生率均高于UD-HSCT组（*P*<0.05）。本研究结果显示，haplo-cord-PBSCT组与UD-PBSCT组的Ⅱ～Ⅳ度、Ⅲ/Ⅳ度急性GVHD发生率差异无统计学意义，慢性GVHD、重度慢性GVHD的累积发生率也没有明显差异。这提示脐带血的加入可能降低了haplo-cord-PBSCT组GVHD的发生率，具体机制还需要进一步的研究。脐带血含有丰富的调节性T细胞（Treg细胞）[23]，Brunstein等[24]的研究显示脐带血Treg细胞的输注能够安全有效地降低脐带血移植患者GVHD的发生率。此外，有研究显示，在haplo-HSCT的急性GVHD小鼠模型中，脐带血中Treg细胞有助于降低GVHD的发生率[25]。

Luo等[13]发现基于G-CSF/ATG方案的haplo-PBSCT组的复发率低于UD-HSCT组（14.2％对21.2％）；Yu等[14]发现基于G-CSF/ATG方案的haplo-PBSCT联合骨髓移植组患者的复发率与UD-HSCT组无明显差异。既往研究表明，不仅单倍体移植物与脐带血均能发挥移植物抗白血病（GVL）作用，能够有效预防疾病复发[26]–[29]，同时脐带血的加入能在不增加GVHD的情况下增强单倍体造血干细胞的GVL效应[6]。Lyu等[30]的研究发现，与UD-HSCT治疗恶性血液病相比，haplo-cord-PBSCT的复发率较低。在本研究中，haplo-cord-PBSCT与UD-PBSCT两组复发率差异无统计学意义，这可能与前者高危患者占比较高有关。

本研究存在一定程度上的局限性。首先，作为一个单中心的回顾性研究，存在一定的误差偏倚，且样本量相对较少也会影响统计的准确性；此外，本研究探究haplo-cord-PBSCT与UD-PBSCT的疗效，缺乏与本中心无脐带血辅助的haplo-HSCT数据的直接比较。

总之，本研究结果表明haplo-cord-PBSCT与UD-PBSCT治疗恶性血液病具有相似的疗效和安全性，可作为恶性血液病的可选治疗方案。

## References

[1] Lu Y, Zhao YL, Zhang JP (2021). Comparable outcomes among unmanipulated haploidentical, matched unrelated, and matched sibling donors in BU-based myeloablative hematopoietic stem cell transplantation for intermediate and adverse risk acute myeloid leukemia in complete remission: a single-center study[J]. Ann Hematol.

[2] Sanz J, Galimard JE, Labopin M (2021). Post-transplant cyclophosphamide containing regimens after matched sibling, matched unrelated and haploidentical donor transplants in patients with acute lymphoblastic leukemia in first complete remission, a comparative study of the ALWP of the EBMT[J]. J Hematol Oncol.

[3] Wieduwilt MJ, Metheny L, Zhang MJ (2022). Haploidentical vs sibling, unrelated, or cord blood hematopoietic cell transplantation for acute lymphoblastic leukemia[J]. Blood Adv.

[4] Wang Y, Liu QF, Xu LP (2015). Haploidentical vs identical-sibling transplant for AML in remission: a multicenter, prospective study[J]. BLOOD.

[5] Kwon M, Bautista G, Balsalobre P (2017). Haplo-cord transplantation compared to haploidentical transplantation with post-transplant cyclophosphamide in patients with AML[J]. Bone Marrow Transplant.

[6] Yang Y, Zhang M, Li M (2022). Unrelated umbilical cord blood can improve the prognosis of haploidentical hematopoietic stem cell transplantation[J]. Stem Cell Res Ther.

[7] Cheng T, Chen Y, Liu Y (2022). Comparison of outcomes of haploidentical peripheral blood stem cell transplantation supported by third-party cord blood versus human leukocyte antigen-matched sibling peripheral blood stem cell transplantation in hematologic malignancy patients[J]. Front Oncol.

[8] Przepiorka D, Weisdorf D, Martin P (1995). 1994 Consensus Conference on Acute GVHD Grading[J]. Bone Marrow Transplant.

[9] Jagasia MH, Greinix HT, Arora M (2015). National Institutes of Health Consensus Development Project on Criteria for Clinical Trials in Chronic Graft-versus-Host Disease: I. The 2014 Diagnosis and Staging Working Group report[J]. Biol Blood Marrow Transplant.

[10] Wang Y, Wang HX, Lai YR (2016). Haploidentical transplant for myelodysplastic syndrome: registry-based comparison with identical sibling transplant[J]. Leukemia.

[11] Huang J, Huang F, Fan Z (2020). Haploidentical related donor vs matched sibling donor allogeneic hematopoietic stem cell transplantation for acute myeloid leukemia and myelodysplastic syndrome aged over 50 years: A single-center retrospective study[J]. Cancer Med.

[12] Sanz J, Galimard JE, Labopin M (2020). Post-transplant cyclophosphamide after matched sibling, unrelated and haploidentical donor transplants in patients with acute myeloid leukemia: a comparative study of the ALWP EBMT[J]. J Hematol Oncol.

[13] Luo Y, Xiao H, Lai X (2014). T-cell-replete haploidentical HSCT with low-dose anti-T-lymphocyte globulin compared with matched sibling HSCT and unrelated HSCT[J]. Blood.

[14] Yu SJ, Fan Q, Sun J (2016). Haploidentical transplantation without in vitro T-cell depletion results in outcomes equivalent to those of contemporaneous matched sibling and unrelated donor transplantation for acute leukemia[J]. Medicine (Baltimore).

[15] Gooptu M, Romee R, St Martin A (2021). HLA-haploidentical vs matched unrelated donor transplants with posttransplant cyclophosphamide-based prophylaxis[J]. Blood.

[16] Zhu XY, Tang BL, Sun ZM (2021). Umbilical Cord Blood Transplantation: Still Growing and Improving[J]. Stem Cells Transl Med.

[17] Theunissen K, Verfaillie CM (2005). A multifactorial analysis of umbilical cord blood, adult bone marrow and mobilized peripheral blood progenitors using the improved ML-IC assay[J]. Exp Hematol.

[18] Anasetti C, Logan BR, Lee SJ (2012). Peripheral-blood stem cells versus bone marrow from unrelated donors[J]. N Engl J Med.

[19] Nagler A, Dholaria B, Labopin M (2020). Bone marrow versus mobilized peripheral blood stem cell graft in T-cell-replete haploidentical transplantation in acute lymphoblastic leukemia[J]. Leukemia.

[20] Lu W, Jin X, Lyu H (2022). A prospective trial comparing haploidentical donor transplantation with cord blood versus HLA-matched sibling donor transplantation for hematologic malignancy patients[J]. Cell Transplant.

[21] Chen J, Wang RX, Chen F (2014). Combination of a haploidentical SCT with an unrelated cord blood unit: a single-arm prospective study[J]. Bone Marrow Transplant.

[22] Huang XJ, Xu LP, Liu KY (2009). Partially matched related donor transplantation can achieve outcomes comparable with unrelated donor transplantation for patients with hematologic malignancies[J]. Clin Cancer Res.

[23] Kim YJ, Broxmeyer HE (2011). Immune regulatory cells in umbilical cord blood and their potential roles in transplantation tolerance[J]. Crit Rev Oncol Hematol.

[24] Brunstein CG, Miller JS, Mckenna DH (2016). Umbilical cord blood-derived T regulatory cells to prevent GVHD: kinetics, toxicity profile, and clinical effect[J]. Blood.

[25] Hao L, Gao L, Chen XH (2011). Human umbilical cord blood-derived stromal cells prevent graft-versus-host disease in mice following haplo-identical stem cell transplantation[J]. Cytotherapy.

[26] Yu S, Huang F, Wang Y (2020). Haploidentical transplantation might have superior graft-versus-leukemia effect than HLA-matched sibling transplantation for high-risk acute myeloid leukemia in first complete remission: a prospective multicentre cohort study[J]. Leukemia.

[27] Chang YJ, Wang Y, Xu LP (2020). Haploidentical donor is preferred over matched sibling donor for pre-transplantation MRD positive ALL: a phase 3 genetically randomized study[J]. J Hematol Oncol.

[28] Zheng CC, Zhu XY, Tang BL (2017). Clinical separation of cGvHD and GvL and better GvHD-free/relapse-free survival (GRFS) after unrelated cord blood transplantation for AML[J]. Bone Marrow Transplant.

[29] Zheng CC, Zhu XY, Tang BL (2015). Comparative analysis of unrelated cord blood transplantation and HLA-matched sibling hematopoietic stem cell transplantation in children with high-risk or advanced acute leukemia[J]. Ann Hematol.

[30] Lyu H, Lu W, Yao J (2020). Comparison of outcomes of haploidentical donor hematopoietic stem cell transplantation supported by third-party cord blood with HLA-matched unrelated donor transplantation[J]. Leuk Lymphoma.

